# Acute viral respiratory infections are not associated with an increased IMMune Age indeX (IMMAX) four weeks after infection: a prospective observational cohort study

**DOI:** 10.1186/s12979-026-00601-8

**Published:** 2026-09-22

**Authors:** Sina Trebing, Bürkan Recep Kalaycik, Maren Claus, Carsten Watzl

**Affiliations:** 1 Department for Immunology, Leibniz Research Centre for Working Environment and Human Factors (IfADo), Ardeystrasse 67, Dortmund, 44139 Germany; 2 Institute for Sport and Sport Science, TU Dortmund University, 44227 Dortmund, Germany

**Keywords:** Immune age, Immune cell, Immunosenescence, Respiratory infection, SARS-CoV-2

## Abstract

**Background:**

The factors that determine how quickly immunosenescence occurs in individuals are not yet fully understood. Viral infections throughout one’s life span are suggested to be a relevant modulator. Latent chronic infections, such as CMV or EBV, occupy immune space and are associated with advanced immunological ageing as measured by biomarkers. The IMMune Age indeX (IMMAX) is a validated surrogate for the IMM-AGE metric based on flow cytometry. It is currently unknown whether light to moderate acute, community-acquired infections with common viruses in Western Europe provoke an increase in biomarkers assessing an individual’s immunological age. Therefore, this study examined IMMAX changes from pre- to post-viral infection, while additionally exploring IMMAX responses during the acute phase of a respiratory illness.

**Methods:**

A prospective observational study was conducted involving a cohort of 77 participants aged between 21 and 65 years. Baseline assessment (t0) included recording demographic data and IMMAX calculation. In the event of symptoms of a respiratory infection, blood samples were taken within the first 72 h (t1), after one week (t2) and after four weeks (t3) for IMMAX follow-up assessment. Viral multiplex PCR was performed at t1 to identify the causative pathogen. Additionally, participants who remained clinically healthy for over 6 months underwent a second IMMAX assessment (t4) and acted as a control group. The primary outcome was the change in IMMAX from t0 to t3, while time- and etiology-dependent differences were evaluated using linear mixed-effects modeling.

**Results:**

Twenty-six participants caught a respiratory infection including human rhinovirus (*n* = 9), SARS-CoV-2 (*n* = 9), parainfluenza virus (*n* = 1) and 7 cases of PCR-negative respiratory illness. Over the confirmed viral infection cohort (*n* = 19), IMMAX values transiently increased during the early acute phase of infection, which was no longer observed after recovery. However, subgroup analysis of all cases of acute respiratory illness (*n* = 26) revealed a remarkable difference: participants with SARS-CoV-2 infection exhibited significantly higher IMMAX values at t1 and lower IMMAX values at t3 compared to baseline, with all nine participants having fully recovered clinically by t3.

**Conclusions:**

This observational cohort study prospectively investigates community-acquired infections with concomitant immune age determination. Mild to moderate viral infections followed by complete recovery were not associated with an increased IMMAX score four weeks after infection, regardless of the underlying etiology. Nevertheless, acute SARS-CoV-2 infections lead to distinct shifts in systemic immune cell proportions, highlighting their immunological demands.

**Supplementary Information:**

The online version contains supplementary material available at https://doi.org/10.1186/s12979-026-00601-8.

## Background

The characteristics of immunosenescence have now been well described. Quantitative shifts in T cell populations include a decrease in naïve T cells in favor of rising memory T cells, as well as reduced T cell receptor (TCR) diversity [1]. Senescent cells cause inflammaging, a state of low-grade systemic inflammation, through their senescence-associated secretory phenotype (SASP). Additionally, metabolic and epigenetic alterations associated with immunosenescence trigger cardiometabolic diseases, neurodegenerative diseases and cancer. The functionality of immune cells involved in host defense is impaired with aging, resulting in an increased susceptibility and severity of infections [1].

The vast majority of community-acquired infections are caused by viruses. In terms of the duration of pathogen activity, viral infections can be classified as either acute or persistent. Following acute infection, adaptive immune responses involving CD4 + helper T cells, CD8 + cytotoxic T cells and plasma cells can eliminate the virus, and generate serotype-specific immunological memory. Common respiratory viruses are the human rhinovirus, which accounts for 30–50% of colds [2], and influenza that cause seasonal waves of infection. Human respiratory syncytial virus (RSV) predominantly causes upper and lower airway infections in childhood. Experimental studies have suggested that acute viral infections may induce cellular senescence: oxidative stress-mediated DNA damage was shown to initiate a senescent phenotype of mononuclear cells during RSV infection [3], while IFN-γ secretion by virus-infected cells may induce cellular senescence via the p53 pathway, at least in endothelial cells [4]. However, whether acute viral infections can accelerate immunosenescence at the systemic level remains unclear.

In contrast, the evidence is much more conclusive with regard to viruses which are not eliminated after primary infection, instead establish lifelong latency. Cytomegalovirus (CMV), a member of the herpesvirus family, typically causes asymptomatic primary infection in immunocompetent hosts but exerts profound long-term effects on the immune system. CMV infection is characterized by the accumulation of late-differentiated effector memory T cells [5]. This phenomenon is referred to as memory inflation. Persistent antigenic stimulation by CMV has been shown to provoke an exhausted state of CD8 + T cells marked by mitochondrial dysfunction and metabolic dysregulation [6]. Increased secretion of proinflammatory cytokines and a reduction of the naïve T cell compartment further resemble hallmarks of immunosenescence. Given its high seroprevalence - affecting 50–90% of adults – CMV has therefore become an important model for studying infection-associated immune aging. In a cohort of 150 healthy individuals, Nicoli et al. (2022) demonstrated that CMV positivity was strongly associated with reduced naïve CD4 + T cell counts, whereas the loss of naïve CD8 + T cells was primarily age-dependent [7]. CMV-positive individuals also exhibited impaired antibody production and CD4 + T cell responses following vaccination [7]. Similarly, a twin study by Yan et al. (2021) identified CMV positivity as a relevant contributor to interindividual immune diversity [8]. Together, these findings indicate that persistent viral infections can substantially shape the immune aging phenotype.

In addition to viral infections, an individual’s so called ‘immunobiography’ [9] is shaped by a range of cumulative lifetime exposures, including diet, physical exercise, the microbiome, combating neoplastic cells, and exposure to other pathogenic entities and vaccinations. The interaction of these immune challenges and (epi)genetic factors modulates the rate of immune aging, thereby explaining the discrepancy between immunological and chronological age [10–12].

Given the central role of T cells in immunosenescence, T cell-based biomarkers have been developed to quantify immunological age. The IMMune Age indeX (IMMAX) is a flowcytometry-based metric that was developed in 2022 as a less complex approximation of the IMM-AGE by Alpert et al., while retaining reasonable accuracy [13, 14]. In a recent evaluation of immune biomarkers, the IMM-AGE, a multimodal biomarker that includes gene expression data in addition to cellular parameters, was rated highly with regard to immunological relevance, temporal association, and predictiveness [15]. The IMMAX is based on five parameters reflecting age-related changes in lymphocyte composition and T cell differentiation: the NK/T cell ratio, CD4^+^/CD8^+^ T cell ratio, memory/naïve CD4^+^ and CD8^+^ T cell ratios, and the proportion of CD28^−^-CD8^+^ T cells. It was established in a working population cohort of 597 volunteers in Germany [13] and has since been applied in various methodological and clinical settings [16–18].

Previous studies have explored immune age biomarkers in the context of infections. In the Dortmund Vital Study (DVS; ClinicalTrials.gov Identifier: NCT05155397), latent Toxoplasma gondii infection was not associated with accelerated immune aging as assessed by IMMAX [17]. The sc-ImmuAging, a single-cell RNA sequencing-based aging clock, disclosed accelerated aging signatures across several immune cell populations, including CD4 + and CD8 + T cells, during acute Covid-19 [19]. The metric IMM-AGE, which formed the basis for IMMAX development, was also applied to COVID-19. Lord et al. recruited 103 recovered patients and were able to show in their linear regression that the IMM-AGE increased with the severity of the previous infection [20]. Collectively, these findings raise the possibility that acute infections caused by COVID-19 and other common viral pathogens may influence biological measures of immune aging, although it remains unclear whether such changes are transient or persist after recovery.

The suitability of the IMMAX metric for longitudinal research has been demonstrated in a five-year follow-up study [18], enabling the assessment of its responsiveness to apparent immunological events over time. In the present study, the impact of an acute viral respiratory infection on the IMMAX was determined. We focused on the pre- to post-infection differences to assess whether recovery from acute infection is accompanied by sustained changes in this biomarker. Given the established role of NK cells, CD4 + T helper cells and CD8 + cytotoxic T cells at different stages of viral infection, we further hypothesized that the IMMAX may be transiently altered during the acute phase. Finally, based on current knowledge of virus-specific immunological and systemic effects, we hypothesized that distinct virus-dependent differences in IMMAX responses may exist.

## Methods

### Study design

Participants were recruited between December 2023 and September 2025 through flyers and the institute’s website. To ensure broad representation of the IMMAX range, at least 25% were recruited from each of three age categories (18–35 years, 35–55 years, > 55 years). At baseline (t0) they had to be free of infections or vaccinations for at least four weeks. Exclusion criteria were chronic-inflammatory or malignant diseases, medication with the potential to influence immune cell count or function, and scheduled participation in drug treatment studies. Participants were asked to avoid any moderate or intensive physical activity for 24 h prior to each assessment in order to eliminate the potential impact of physical activity on immune cell counts. The study was approved by the ethics committee of the Leibniz Research Centre for Working Environment and Human Factors (#245).

#### t0

After obtaining written informed consent, demographic (sex, date of birth, height, weight) and anamnestic (medication, supplements, date of last infection and vaccination, and hours since last exercise) information was collected. Then, 2.7 ml of blood was drawn from a cubital vein and collected in EDTA tubes. The subjects agreed to inform the study staff by phone or email as soon as possible if they fell ill.

#### t1-3

Follow-up appointments were arranged for within the first 72 h (t1) of an acute respiratory infection affecting the upper and/or lower respiratory tract, one week later (t2) and four weeks later (t3). Each of them took place at the same time (± 30 min) as t0 in order to rule out circadian fluctuations, although a subsequently published study reported stable values throughout the day [16]. At each assessment, patients completed a symptom questionnaire and had 2.7 ml of blood drawn. At t1, a nasopharyngeal swab was taken. Multiplex PCR to detect influenza A and B, SARS-CoV-2, RSV, parainfluenza, adenovirus, human rhinovirus, and metapneumovirus was performed at the Institute for Medical Lab Diagnostics in Bochum (IML), Germany. The PCR results were provided in written form by IML as positive or negative for each virus, for SARS-CoV-2, the cycle threshold (Ct) value was additionally reported. Participants with clinical symptoms of bacterial inflammation were excluded. These indications were assessed by a physician and included persistent fever despite a negative PCR result, purulent pharyngitis, increasing cough with purulent sputum, or auscultatory findings suggestive of pneumonia at t1 and t2.

#### t4

All participants who had completed the baseline assessment at t0 were eligible for a follow-up IMMAX assessment after six months (t4). Participants who developed any infection during the six-month follow-up period were excluded from the t4 reference group. Individuals without any reported infection during the six-months period were assessed at t4 and comprised the control group (CG), which served as a reference for the natural six-months course of the IMMAX in the absence of a reported intervening infection. Health status was assessed bimonthly by self-report.

### Sample size calculation

The sample size was determined a priori using G*Power (version 3.1.9.6, Heinrich-Heine-Universität Düsseldorf, Germany). Due to the lack of published data allowing specific estimation of the effect of a viral respiratory infection on immune-age indices, an effect size of *f* = 0.3 was assumed. Based on repeated-measures (rm) ANOVA with within-subject factors, four measurement time points, a significance level of 0.05, and a power of 0.8, a minimum sample size of 17 participants was calculated. As follow-up measurements at t1-t3 depended on the development of a viral infection, an anticipated dropout rate of approximately 75% was considered, resulting in a planned minimum sample size of 70 participants.

### Flow cytometry and IMMAX calculation procedure

Within one hour after blood collection, 100 µl of the EDTA-anticoagulated blood was directly immunostained with a combination of seven antibodies (BV421 anti-CD4 (clone RPA-T4, Cat. 562424, BD Horizon™), BV510 anti-CD3 (clone UCHT1, Cat. 568556, BD Horizon™), BB515 anti-CD8 (clone RPA-T8, Cat. 564526, BD Horizon™), PerCP-Cy™ 5.5 anti-CD28 (clone CD28.2, Cat. 560685, BD Pharmingen™), PE anti-CCR7 (CD197) (clone 3D12, Cat. 552176, BD Horizon™), PE-Cy™5 anti-CD56 (clone B159, Cat. 555517, BD Pharmingen™), Alexa Fluor^®^ 700 anti-CD45RA (clone HI100, Cat. 560673, BD Pharmingen™), and a Fixable Viability Dye eFluor™ 780 (Cat. 65-0865-14, eBioscience™) in Brilliant Stain Buffer (Cat. 566349, BD Horizon™). The antibody master mix was freshly prepared on each measurement day, using same antibody batches throughout the study period. After incubation for 30 min at room temperature, red blood cells were lysed with 1 ml of FACS Lysing Solution (BD Biosciences). Samples were centrifuged, supernatant was discarded, and pellets were resuspended in 250 µl of flowcytometry buffer (PBS + 2% FCS). 30.000 lymphocytes were acquired by FACS DIVA Software using BD LSRFortessa™ Cell Analyzer. Data were analyzed using FlowJo™ v10.10 Software (BD Life Sciences). Gating strategy as published recently [17] was used to determine the following relative cell frequencies: NK/T cell ratio, CD4^+^/CD8^+^ T cell ratio, memory/naïve CD4^+^ and CD8^+^ T cell ratios, CD28^−^-CD8^+^ T cells. IMMAX values were calculated from the relative cell frequencies as described before and converted into equivalent years of life (EYOL) and an age gap (= EYOL based on the IMMAX – chronological age) [18]. An Excel sheet containing the equations and coefficients used for IMMAX calculation is provided in the supplementary material.

### Statistical analysis

Statistical analyses were performed using GraphPad Prism 10 version 11.1.0 for macOS X (Boston, Massachusetts, USA) and Python (version 3.13). After testing for normal distribution using the Shapiro-Wilk test, a Spearman correlation analysis was performed on the total population’s (*n* = 34) chronological and immunological age at t0 as an internal consistency check of baseline IMMAX data. Although sample size calculation was based on a repeated-measures ANOVA, a mixed-effects model was used for final analysis to account for missing repeated measurements and make more efficient use of available observations. The prespecified primary outcome was the change in IMMAX from t0 to t3 in participants with PCR-confirmed viral infections. Geisser-Greenhouse correction was used and post hoc comparisons of each follow-up time point with baseline were performed using Dunnett’s multiple comparisons test. Model assumptions were assessed by inspection of Q-Q plots and residual plots. Baseline characteristics with regard to demographic data, anthropometric data, baseline IMMAX, and vaccination status were summarized descriptively across the three acute respiratory illness groups and the healthy control group. An exploratory subgroup analysis according to etiology (human rhinovirus, SARS-CoV-2, or other acute respiratory illness) was performed using a mixed-effects model with Dunnett’s multiple comparisons test. In addition to analyses of the overall IMMAX across all participants with acute respiratory illness, its five single components were analyzed over time. P values were adjusted for multiple testing where applicable. Level of significance (α) was set to 0.05.

## Results

**Fig. 1:**
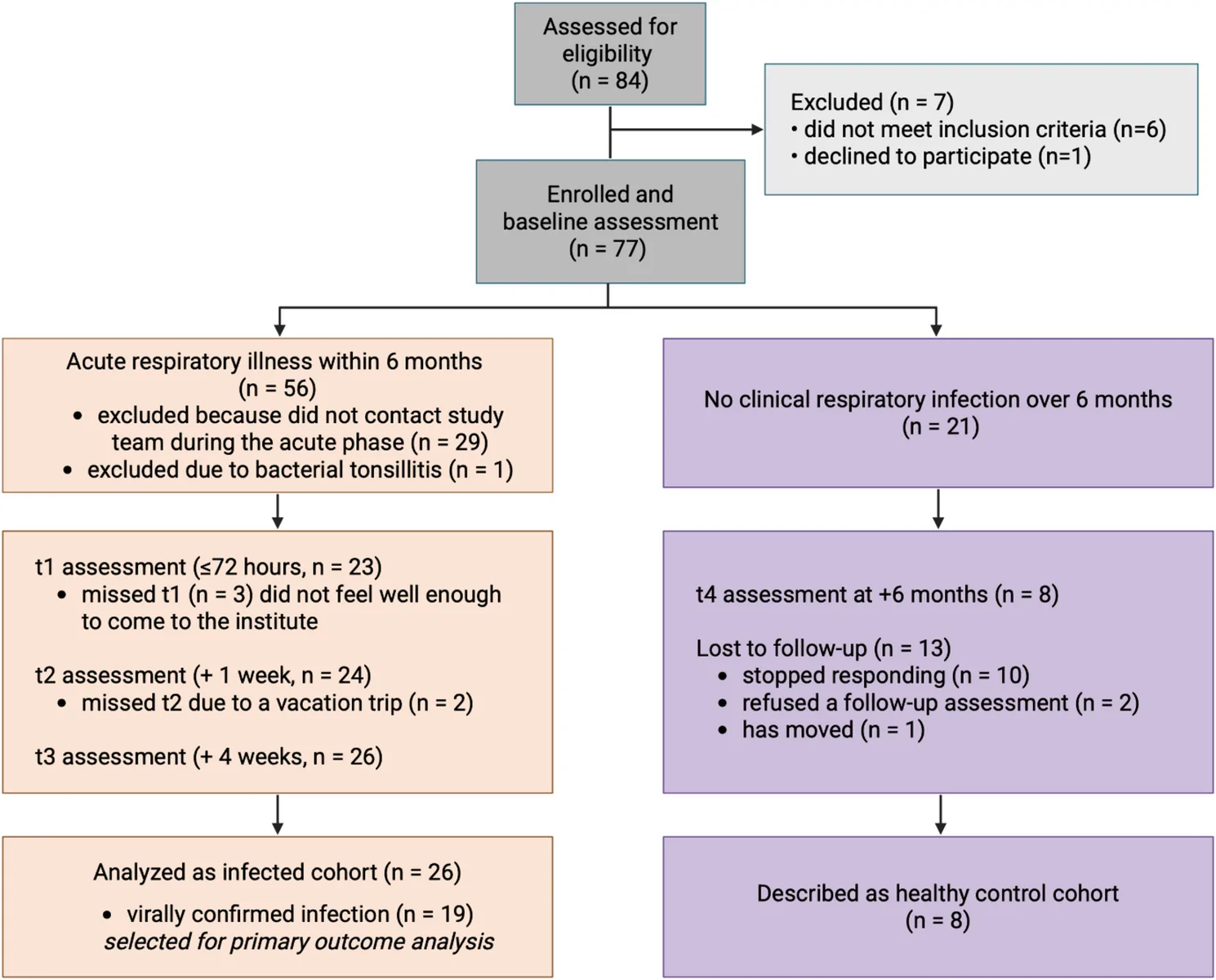
Participant flow diagram (Created in BioRender. Watzl, C. (2026) https://BioRender.com/q4t1qxd)

Of the 77 participants recruited (mean age: 40.8 years; sex ratio (F/M):49/28), 27 developed a respiratory infection within six months and contacted the study team within the first 72 h of symptom onset. One participant (*n* = 1) was excluded from this cohort due to a confirmed *Staphylococcus aureus* infection that had been diagnosed by a general practitioner outside the study setting. The healthy control assessment was completed in eight participants who had remained free of clinical infection for at least six months (t4 = t0 + 6–7 months). The flow of participants is illustrated in Fig. 1.

Viral multiplex PCR revealed infections with human rhinovirus (*n* = 9), SARS-CoV-2 (*n* = 9), and parainfluenza virus (*n* = 1). These 19 participants (mean age: 34.9 years; (F/M):13/6) constituted the confirmed viral infection cohort. In addition, seven participants were clinically symptomatic but tested negative for the eight viruses included in the standard multiplex panel and were therefore classified as having acute respiratory illness of unknown etiology. Thus, the total infected cohort comprised 26 participants with acute respiratory illness. Of these, 15 had an upper respiratory tract infection and 11 had a lower respiratory tract infection, distributed across all etiological groups (upper/lower: human rhinovirus, *n* = 6/3; SARS-CoV-2, *n* = 4/5; other etiologies, *n* = 5/3). For analysis, the single case of parainfluenza infection was grouped with the seven cases of of acute respiratory illness of unknown etiology into the “other etiologies” category. Baseline characteristics of participants, summarized by subgroup, are shown in Table 1.

**Table 1:** Descriptive characteristics of the study participants grouped by human rhinovirus, SARS-CoV-2-virus, other etiologies of acute respiratory illness, or healthy control group

	Human Rhinovirus *n* = 9			SARS-CoV-2 *n* = 9			Other etiologies *n* = 8			Healthy Control *n* = 8		
sex (F / M)	6 / 3			6 / 3			7 / 1			6 / 2		
	Min	Max	Mean (SD)	Min	Max	Mean (SD)	Min	Max	Mean (SD)	Min	Max	Mean (SD)
age (years)	26	65	34.3 (12.00)	21	55	33.3 (11.60)	21	55	37.5 (14.55)	20	70	41.8 (16.19)
BMI (kg/m^2^)	18.7	35.6	25.9 (4.59)	20.3	33.2	25.0 (4.07)	19.1	36.1	23.0 (5.86)	21.8	29.1	24.5 (2.91)
baseline IMMAX	0.31	0.75	0.42 (0.14)	0.21	0.73	0.40 (0.15)	0.26	0.45	0.38 (0.07)	0.19	0.54	0.41 (0.14)
vaccination status (number of vaccinated subjects (in %))												
SARS-CoV-2 (≥ 3 doses)	9 (100%)			8 (88.8%)			6 (75%)			6 (75%)		
influenza (current season)	2 (22.2%)			1 (11.1%)			2 (25%)			2 (25%)		
pneumococcal	2 (22.2%)			1 (11.1%)			0 (0%)			1 (12.5%)		

As expected [16, 18], , the IMMAX values of the whole study cohort (*n* = 34) showed a significant correlation with chronological age at baseline (Spearman *r* = 0.463, *p* = 0.006).

**Fig. 2:**
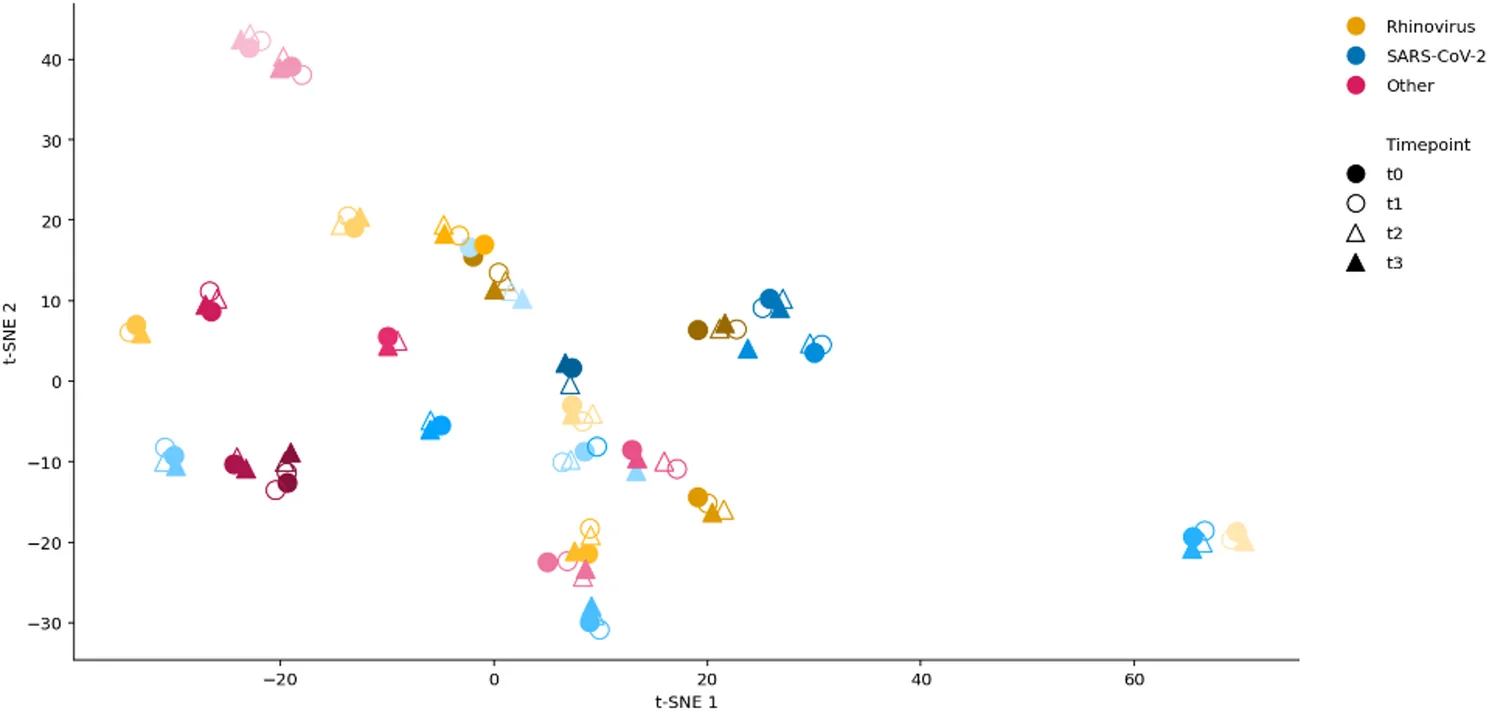
t-distributed stochastic neighbor embeddings (t-SNE) based on log2-transformed relative frequencies of 11 correlated features (CD3^−^CD56^+^ lymphocytes (NK cells), CD3^+^CD56^−^ lymphocytes (T cells), CD4^+^ T cells, CD4^+^CD45RA^−^CD197^+^ T cells, CD4^+^CD45RA^+^CD197^+^ T cells, CD4^+^CD45RA^−^CD197^−^ T cells CD8^+^ T cells, CD8^+^CD45RA^−^CD197^+^ T cells, CD8^+^CD45RA^+^CD197^+^ T cells, CD8^+^CD45RA^−^CD197^−^ T cells, and CD8^+^CD28^−^ T cells) at the sample level. Each icon refers to a sample; colors represent the participant ID and etiology (yellow-orange for human rhinovirus, blue for SARS-CoV-2, and red for other etiologies), while icon form indicates measurement time point. Samples with similar characteristics across the 11 features are positioned closer together

To explore whether acute respiratory illnesses were associated with distinct immune cell feature profile, an exploratory t-SNE analysis was performed. As shown in Fig. 2, samples from the same participant clustered together, irrespective of infection history. A similar clustering pattern of the relative frequencies of the eleven features that make up the IMMAX was observed among healthy controls over the 6-month interval (see supplementary Figure S1).

**Fig. 3:**
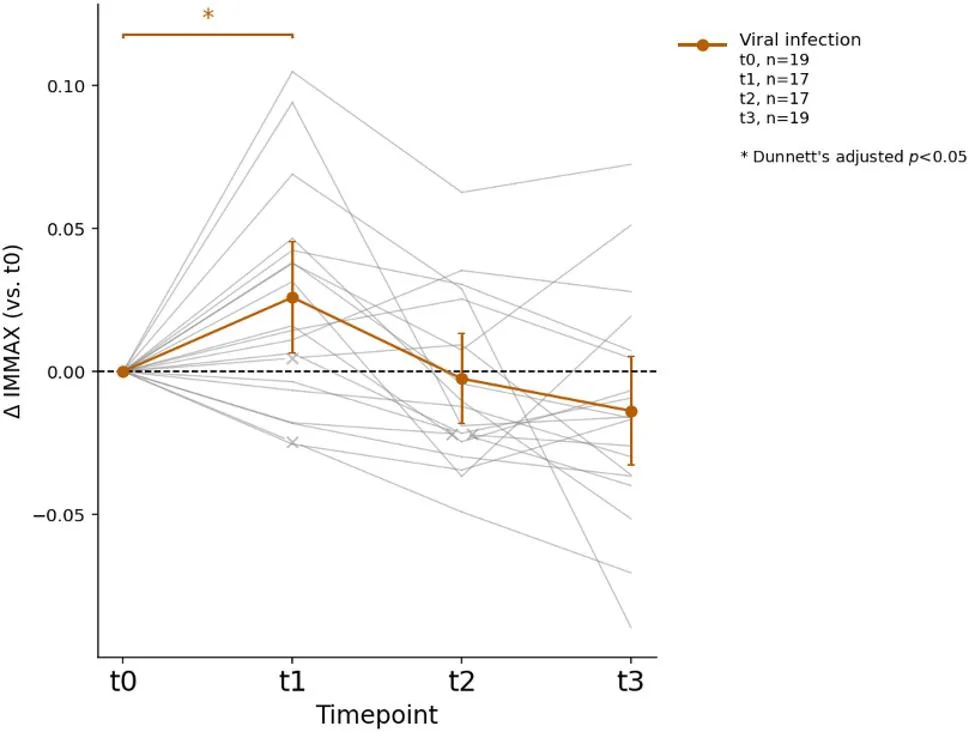
Course of the IMMAX in participants with PCR-confirmed viral infections. Individual IMMAX trajectories are shown in grey lines, with each line representing one participant. The orange line indicates the mean change in IMMAX from baseline at each time point, with error bars representing 95% CI. Crosses indicate missing measurements at the respective time points (t1, *n* = 2; t2, *n* = 2)

We next evaluated the primary outcome, defined as the change in IMMAX from baseline to t3 in the cohort with virologically confirmed infection. Estimated mean difference (t3-t0) was − 0.014 (95% CI -0.037–0.009; adjusted *p* = 0.310), indicating no statistically significant change (Fig. 3). A sensitivity analysis excluding the four participants with missing intermediate measurements yielded comparable results.

**Fig. 4:**
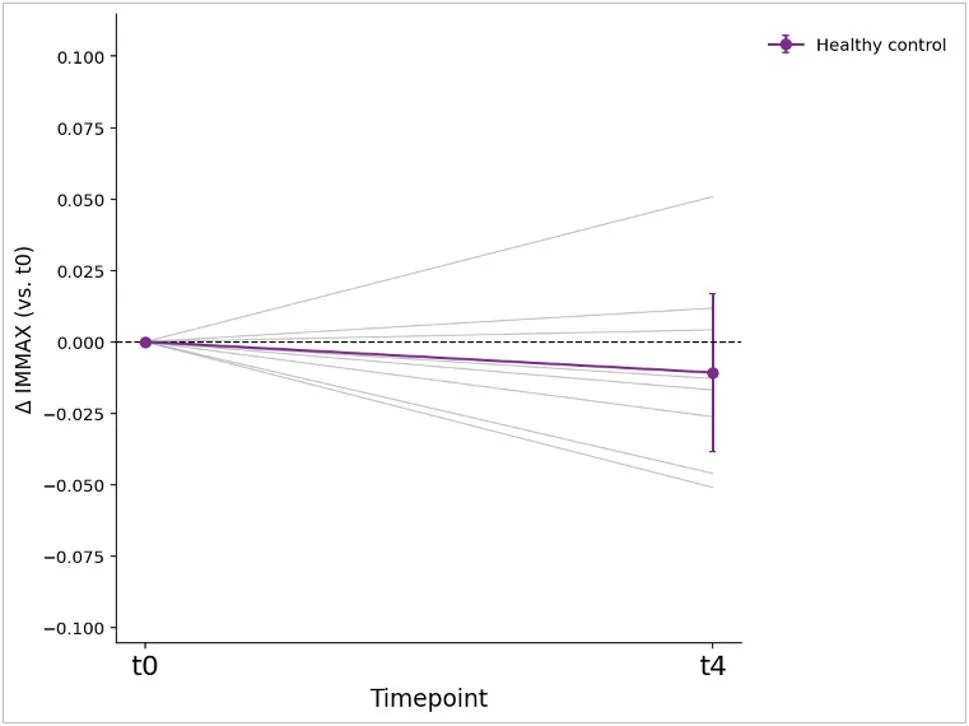
Course of the IMMAX in healthy control group. Individual IMMAX trajectories are shown in grey lines, with each line representing one participant (*n* = 8). The purple line indicates the mean change in IMMAX from baseline, with error bar representing the 95% CI

In healthy controls, the course from baseline to t4, reflecting the natural course in the absence of infection, showed a comparable pattern descriptively (Fig. 4).

**Fig. 5:**
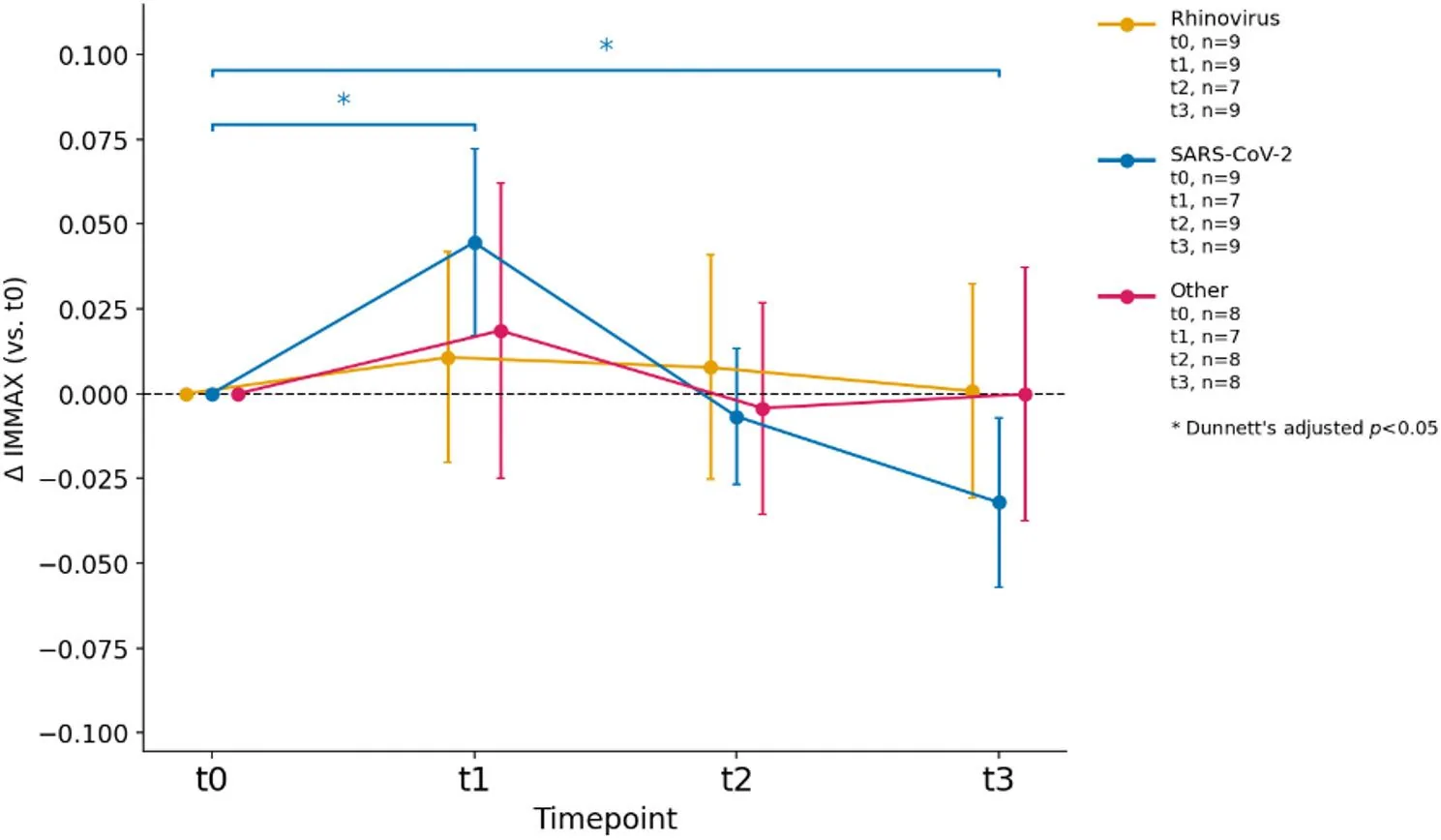
Exploratory subgroup analysis: Mean Δ IMMAX values relative to baseline (t0) with 95% CIs over time, stratified by infection etiology: orange, human rhinovirus infection; blue, SARS-CoV-2 infection; red, other acute respiratory illnesses. Sample sizes at each time point are provided in the legend. A significant time effect detected by the mixed-effects model was followed by Dunnett’s multiple comparisons test, comparing each time point with baseline (t0). Significant within-group differences from baseline, observed exclusively in the SARS-CoV-2 group, are indicated (t1 – t0, *p* = 0.0185*; t3 – t0, *p* = 0.0418*). *P* values are Dunnett-adjusted

To further characterize the temporal dynamics of the IMMAX, the mixed-effects model revealed a significant effect of time, with subsequent Dunnett-adjusted post-hoc analysis showing significantly higher IMMAX values in the early acute phase (t1) compared with baseline (estimated mean difference: 0.026, 95% CI 0.002–0.050, adjusted *p* = 0.035) (Fig. 3). This finding in the cohort with virologically confirmed infection was complemented by an exploratory subgroup analysis (rhinovirus/SARS-CoV-2/other acute respiratory illness) to detect etiology-dependent effects. Interestingly, the time course was distinct in the SARS-CoV-2 group, with increased IMMAX values at t1 (d0-3), followed by decreasing values at t2 (d7) and t3 (d28). IMMAX values at both t1 and t3 differed significantly from baseline (t1-t0, mean diff. 0.044, 95% CI 0.009–0.079, adjusted *p* = 0.019; t3-t0, mean diff. -0.032, 95% CI -0.001–-0.063, adjusted *p* = 0.041). In contrast, the two other groups exhibited relatively stable IMMAX values. These findings are illustrated in Fig. 5.

**Fig. 6:**
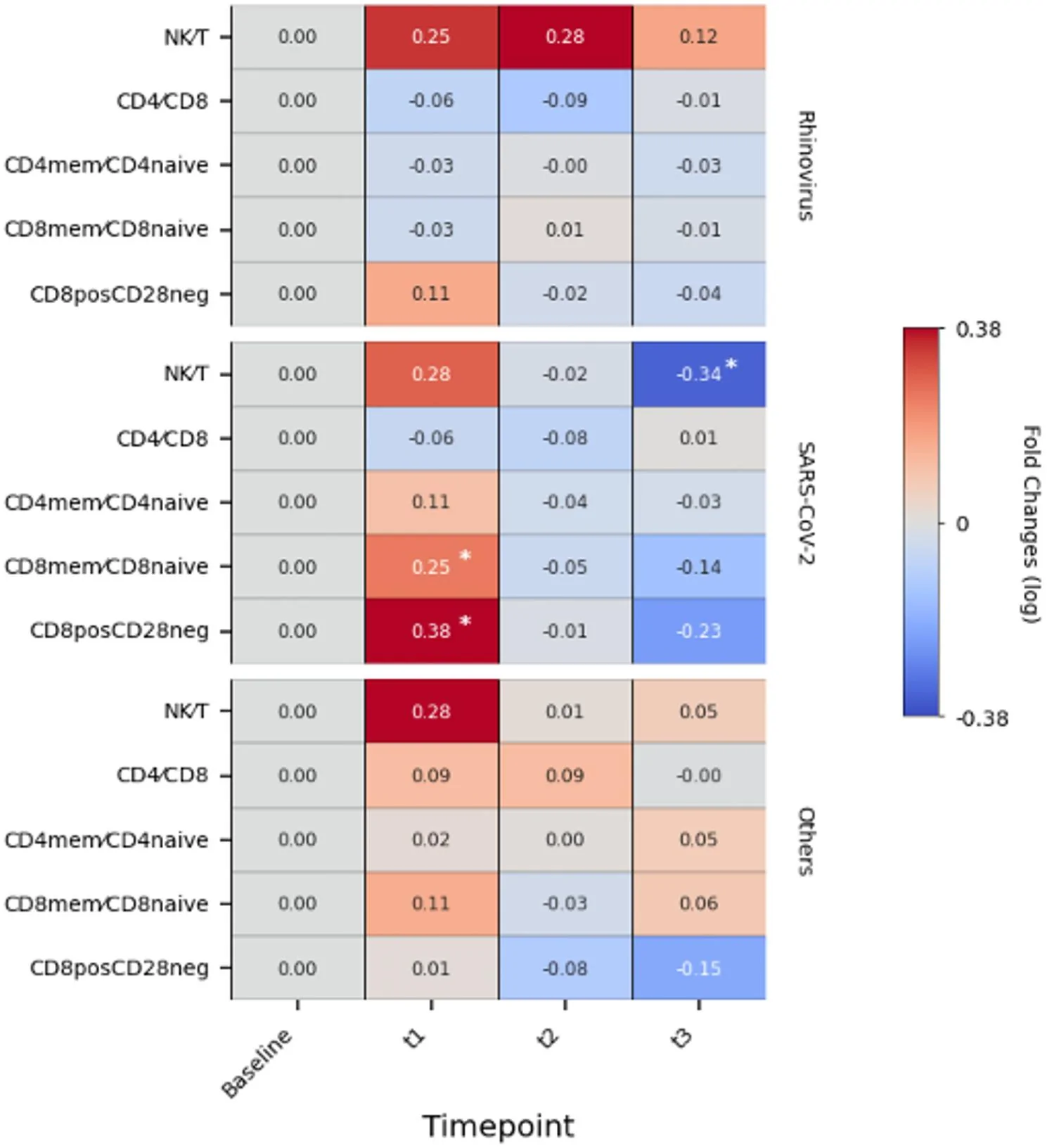
Heatmap showing the kinetics of the IMMAX’s single components throughout the infection period compared to baseline (t0). The three vertical maps indicate the different etiological subgroups. Raw ratios were calculated using FlowJo™ v10 Software and log-fold changes performed. Asterisks indicate significant differences from t0 identified by mixed-effects modeling of logarithmic ratios or logit-transformed CD28^−^CD8^+^ T cell frequencies, respectively, followed by Dunnett’s multiple comparisons test (*p < 0.05)

To evaluate the changes in the five IMMAX components that contributed to the time-dependent variation, a heatmap was constructed showing the deviations from baseline (Fig. 6). Given the observed differences in the longitudinal analysis, the exploratory analysis was subsequently performed separately for each subgroup. Descriptively, the NK/T ratio was elevated at t1 by a similar magnitude across all three subgroups. In participants with human rhinovirus infection, it remained elevated compared with baseline until t2, whereas it had already returned to baseline levels by t2 in participants with SARS-CoV-2 infection and other respiratory illnesses. At t3, a decrease in the NK/T ratio was observed exclusively following SARS-CoV-2 infection and was statistically significant compared with baseline (adjusted *p* = 0.049).

The percentage of CD28^−^-CD8^+^ T cells increased most strongly during the early phase of SARS-CoV-2 infection (t1), After recovery, however, the percentage decreased below baseline levels. A similar pattern was observed for the memory/naïve CD8^+^ ratio in the SARS-CoV-2 group. For both parameters, the difference between t1 and t0 was statistically significant (adjusted *p* = 0.032 for the memory/naïve CD8^+^ ratio and *p* = 0.019 for the CD28^−^-CD8^+^ T cell frequency). A trend towards lower CD28^−^-CD8^+^ T cell percentages after recovery was also observed in participants with other acute respiratory illnesses, although this was not preceded by an increase.

Collectively, significant deviations in the three IMMAX components NK/T ratio, CD28^−^-CD8^+^ T cells, and memory/naïve CD8^+^ ratio, were observed exclusively in the context of an acute SARS-CoV-2 infection.

## Discussion

The present observational study examined the temporal dynamics of the IMMAX score during naturally acquired respiratory infections. Overall, acute viral infections were associated with transient alterations in the IMMAX, while the primary endpoint – the estimated change from t0 to t3 – did not reach statistical significance. Therefore, our data indicate that acute viral respiratory infections do not result in a sustained increase in the IMMAX after recovery.

Notably, SARS-CoV-2 infection induced a distinct short-term increase in IMMAX values in the early acute phase, followed by decrease at four weeks after. The transient increase during the early acute phase (t1) may reflect short-term alterations in immune cell profiles due to cellular activation and differentiation of lymphocytes associated with the acute viral response, rather than expressing an aggravation of immunosenescence. Thus, uncomplicated respiratory infections that resolve completely within four weeks do not appear to induce lasting alterations in the IMMAX. In contrast, CMV seropositivity was found to significantly increase the baseline IMMAX in both female and male participants of a SARS-CoV-2 vaccination efficacy study (unpublished data from the Dortmund Vital Study, Germany). These findings suggest that the distinct immunological footprints of acute and latent infections may be reflected in the differential IMMAX responsiveness.

Despite its pragmatic and limited marker panel, the IMMAX reflected immunological patterns associated with SARS-CoV-2 infection in the exploratory analysis, suggesting that it may be sensitive to infection-related immune perturbations. According to our analysis, shifts in IMMAX components were particularly evident among CD8^+^ T cells, namely in the memory/naïve ratio and the frequency of senescent CD28^−^CD8^+^ T cells, which are well-established features associated with immunosenescence [1, 21]. In contrast, rhinovirus infection did not show comparable alterations in either IMMAX score or its individual components, even though symptom questionnaires and physical examination revealed no differences in subjective disease severity between the two groups.

The IMMAX deflection observed in the SARS-CoV-2 group appeared to be reversible and resolved in parallel with symptom recovery, further supporting a transient nature of these changes. These results are consistent with those by Li et al. who applied their sc-ImmuAging in acute Covid-19 and revealed immunological age acceleration during infection and normalization upon recovery. Two independent approaches for assessing immune age – single-cell RNA sequencing and flow cytometry – thus point to similar dynamics.

Not only did the increase level off here, but IMMAX values also decreased below baseline at t3. As only relative proportions were assessed, the findings do not allow conclusions regarding cellular expansion and contraction. The observed changes in NK/T ratio and, descriptively, in the CD8^+^ T cell compartment may nevertheless have contributed to this pattern and could potentially reflect a transient increase in less differentiated cell populations. Whether the early increase in IMMAX persists in patients with post-COVID syndrome, and whether the decrease below baseline observed after recovery is similarly present in this population, remains an open question. Future studies could address this research area.

When classifying the results in line with the current state of knowledge on SARS-CoV-2 and biomarkers for immunological ageing, disease severity must be taken into account. Our cohort did not include high-risk individuals, and accordingly, the COVID-19 cases were all mild to moderate, with no need for medical consultation. Prior research found early expansion of activated NK cells and that the effect on NK cells correlates with disease severity of COVID-19 [22, 23]. Consequently, one can only speculate that, due to the mild courses of the disease, no effect on the NK/T cell ratio during the early phase that could be distinguished from that of human rhinovirus was observable. It was only the subsequent drop in the NK/T cell ratio following recovery that was a characteristic unique to the COVID-19 group here. As absolute cell counts are not part of the IMMAX method, changes in NK/T ratio cannot be attributed to absolute changes in either NK or T cell numbers and should therefore be interpreted as relative alterations in immune cell composition.

The scImmu Aging, a cell-type specific transcriptomic analysis, revealed the most pronounced age acceleration in monocytes during acute Covid-19 – a population not directly included in IMMAX calculations but already recognized as a central mediator of disease severity due to its role in systemic inflammation [24]. Similarly, they found normalized aging signature after recovery. The immunological and inflammatory processes triggered during acute SARS-CoV-2 infection may promote features of inflammaging and immunosenescence.

Although t-SNE is an exploratory visualization technique rather than a statistical method, the close clustering of intraindividual time points may indicate the presence of a stable individual immunological signature. Notably, these clusters remained largely preserved even during SARS-CoV-2 infection, despite the transient increase observed in the overall IMMAX score.

Some limitations of the present study should be considered. The etiological subgroups, which consisted of eight to nine participants, were rather small. Due to the heterogeneity of the third group, comprising a case of parainfluenza virus infection as well as PCR-negative, symptomatic cases suggestive of infections with pathogens not included in the PCR panel, we could not analyze the subgroups in greater detail.

The use of symptomatic medication during the infection represents a potential influencing factor. Some participants received symptomatic treatment, including non-steroidal anti-inflammatory drugs such as ibuprofen, which may modulate immune responses. Although medication use was recorded, its potential influence on the IMMAX was not assessed separately and therefore cannot be excluded.

In addition, asymptomatic viral infections among participants classified as healthy controls cannot be reliably excluded, as PCR testing was not routinely performed in asymptomatic individuals. Thus, undetected infections may have occurred during the follow-up period, while the observed temporal fluctuations in the IMMAX (Fig. 4) also indicate intra-individual variability in the absence of reported respiratory symptoms.

Here, we focused on viral infections. Nevertheless, an anecdotal observation was noted: One participant reported sore throat as respiratory symptom. The viral PCR test was negative, but a subsequent external microbiological throat swab revealed a bacterial tonsillitis with Staphylococcus aureus. Although this participant was excluded from the analyses, it is worth noting that the IMMAX course was very different from those of viral infections. Even though the participant had clinically recovered by t3 following antibiotic treatment, the IMMAX score remained high. Starting with a baseline IMMAX of 0.30, the peak was reached at t_1_ with 0.70. After four weeks, the IMMAX had only dropped to 0.53 (Fig. S2). This isolated case suggests that non-viral infections may influence IMMAX dynamics differently and deserve further investigation in future studies.

The four-week follow-up period does not allow us to draw conclusion on long-term changes in IMMAX. Our analysis was focused on the acute phase to capture infection-associated changes, as substantial changes in T cell differentiation and the establishment of memory populations occur during this period. Following experimental RSV infection, Jozwik et al. demonstrated marked changes in virus-specific CD8^+^ T cell response during the first days after infection, with proliferation peaking around day 10 and no evidence of ongoing proliferation by day 28 [25]. Nevertheless, longer-term longitudinal studies with sampling several months after infection will be required to determine whether respiratory viral infections induce delayed alterations in markers of immunosenescence.

## Conclusions

In summary, our findings demonstrate that the IMMAX score is largely stable within individuals with an acute viral infection, with a transient increase during the acute phase that was no longer evident shortly after clinical recovery. SARS-CoV-2 infection, however, induced a distinct shift driven primarily by CD8-related components beyond symptom recovery. Thus, differences of immunological response to respiratory infections of different etiologies are reflected in IMMAX measurements. Assessments may therefore be influenced by acute symptomatic infection and should be interpreted with caution during this period. 

## Supplementary Information

Supplementary Material 1.

Supplementary Material 2.

Supplementary Material 3.

Supplementary Material 4.

## Data Availability

The datasets used and/or analyzed during the current study are available from the corresponding author upon request.

## Abbreviations

BMI: Body mass index
CI: Confidence interval
CMV: Cytomegalie virus
EBV: Epstein-Barr virus
EDTA: Ethylenediaminetetraacetic acid
EYOL: Equivalent years of life
IMMAX: Immune Age Index
NK: Cellnatural killer cell
SARS-CoV-2: Severe acute respiratory syndrome coronavirus type 2
