# Supplementary material for: Acute viral respiratory infections are not associated with an increased IMMune Age indeX (IMMAX) four weeks after infection: a prospective observational cohort study
Source: Immun Ageing. 2026 Sep 22;23:36. doi: 10.1186/s12979-026-00601-8 (PMC13595608; doi:10.1186/s12979-026-00601-8)
Supplement: Supplementary file 1 [file 12979_2026_601_MOESM1_ESM.docx]

| No. | t0 | t1 | t2 | t3 | t4 | Etiology of acute respiratory illness  1 = human rhinovirus  2 = SARS-CoV-2  3 = other acute respiratory illness  4 = healthy control |
| --- | --- | --- | --- | --- | --- | --- |
| 01 | 0,321 | 0,426 | 0,384 | 0,394 |  | 1 |
| 02 | 0,37 | 0,363 | 0,358 | 0,34 |  |  |
| 03 | 0,337 | 0,319 | 0,307 | 0,3 |  |  |
| 04 | 0,386 | 0,397 | 0,422 | 0,414 |  |  |
| 05 | 0,386 | 0,36 | 0,351 | 0,369 |  |  |
| 06 | 0,537 | 0,518 |  | 0,51 |  |  |
| 07 | 0,747 | 0,743 |  | 0,707 |  |  |
| 08 | 0,392 | 0,43 | 0,4 | 0,443 |  |  |
| 09 | 0,314 | 0,328 | 0,339 | 0,319 |  |  |
| 10 | 0,38 |  | 0,33 | 0,309 |  | 2 |
| 11 | 0,314 | 0,352 | 0,31 | 0,298 |  |  |
| 12 | 0,32 | 0,362 | 0,35 | 0,327 |  |  |
| 13 | 0,31 |  | 0,319 | 0,274 |  |  |
| 14 | 0,425 | 0,431 | 0,403 | 0,415 |  |  |
| 15 | 0,422 | 0,491 | 0,451 | 0,332 |  |  |
| 16 | 0,211 | 0,305 | 0,192 | 0,195 |  |  |
| 17 | 0,465 | 0,481 | 0,441 | 0,458 |  |  |
| 18 | 0,725 | 0,771 | 0,714 | 0,673 |  |  |
| 19 | 0,434 | 0,465 | 0,397 | 0,453 |  | 3 |
| 20 | 0,452 | 0,431 | 0,46 | 0,46 |  |  |
| 21 | 0,389 | 0,352 | 0,333 | 0,294 |  |  |
| 22 | 0,42 | 0,499 | 0,461 | 0,453 |  |  |
| 23 | 0,326 |  | 0,305 | 0,303 |  |  |
| 24 | 0,255 | 0,292 | 0,309 | 0,304 |  |  |
| 25 | 0,411 | 0,384 | 0,403 | 0,401 |  |  |
| 26 | 0,322 | 0,389 | 0,306 | 0,339 |  |  |
| 27 | 0.54 |  |  |  | 0.527 | 4 |
| 28 | 0.532 |  |  |  | 0.537 |  |
| 29 | 0.250 |  |  |  | 0.233 |  |
| 30 | 0.535 |  |  |  | 0.486 |  |
| 31 | 0.187 |  |  |  | 0.237 |  |
| 32 | 0.498 |  |  |  | 0.448 |  |
| 33 | 0.352 |  |  |  | 0.325 |  |
| 34 | 0.416 |  |  |  | 0.427 |  |

**Supplementary Table 1.** Individual IMMAX values in participants with and without acute respiratory illness. The table provides the individual data underlying the longitudinal analyses presented in the manuscript.
