# Supplementary material for: Acute viral respiratory infections are not associated with an increased IMMune Age indeX (IMMAX) four weeks after infection: a prospective observational cohort study
Source: Immun Ageing. 2026 Sep 22;23:36. doi: 10.1186/s12979-026-00601-8 (PMC13595608; doi:10.1186/s12979-026-00601-8)
Supplement: Supplementary file 2 [file 12979_2026_601_MOESM4_ESM.docx]

Figure S1. t-distributed stochastic neighbor embeddings (t-SNE) with healthy controls (n=8) based on the features percentage of CD3-CD56+ lymphocytes (NK cells), CD3+CD56- lymphocytes (T cells), CD4+ T cells, CD4+CD45RA-CD197+ T cells, CD4+CD45RA+CD197+ T cells, CD4+CD45RA-CD197- T cells CD8+ T cells, CD8+CD45RA-CD197+ T cells, CD8+CD45RA+CD197+ T cells, CD8+CD45RA-CD197- T cells, and CD8+CD28- T cells. Each icon refers to a sample, the colors represent a single ID, and icon form indicates measurement timepoint. Data points that share similar characteristics across the eleven features show overlapping clusters.

Figure S2. Anecdotal presentation of the IMMAX course over time in a single excluded participant with a bacterial infection (n = 1), shown in comparison with the mean IMMAX course of participants with confirmed viral infections included in the main study (n = 19).
